# A prospective observational cohort study of lenvatinib as initial treatment in patients with BCLC-defined stage B hepatocellular carcinoma

**DOI:** 10.1186/s12885-022-09625-x

**Published:** 2022-05-07

**Authors:** Satoshi Kobayashi, Taito Fukushima, Makoto Ueno, Satoshi Moriya, Makoto Chuma, Kazushi Numata, Kota Tsuruya, Shunji Hirose, Tatehiro Kagawa, Nobuhiro Hattori, Tsunamasa Watanabe, Kotaro Matsunaga, Michihiro Suzuki, Haruki Uojima, Hisashi Hidaka, Chika Kusano, Motoko Suzuki, Manabu Morimoto

**Affiliations:** 1 Department of Gastroenterology, Hepatobiliary and Pancreatic Medical Oncology Division, Kanagawa Cancer Center, Nakao 2-3-2, Asahi-ku, Yokohama, Kanagawa 241-8515 Japan; 2 Gastroenterological Center, Yokohama City University Medical Center, Yokohama, Kanagawa Japan; 3 Department of Gastroenterology, Tokai University School of Medicine, Isehara, Kanagawa Japan; 4 Division of Gastroenterology and Hepatology, St. Marianna University School of Medicine, Kawasaki, Kanagawa Japan; 5 Department of Gastroenterology, Kitasato University School of Medicine, Sagamihara, Kanagawa Japan; 6 Department of Data Science, National Cancer Center Hospital East, Kashiwa, Chiba Japan

**Keywords:** Hepatocellular carcinoma, Intermediate stage, Substage, Lenvatinib, TACE

## Abstract

**Background:**

Transarterial chemoembolization (TACE) is the standard treatment for intermediate stage hepatocellular carcinoma (HCC) (Barcelona Clinic Liver Cancer [BCLC] B). However, it often leads to a poor prognosis and decreased hepatic function especially in patients with BCLC substage B2. Lenvatinib (LEN) was demonstrated to be efficacious in these patients in the REFLECT phase 3 trial. We therefore aimed to evaluate the efficacy and safety of LEN as a first-line treatment for the patients with HCC at BCLC substage B2.

**Methods:**

This prospective observational study used LEN in TACE-naïve patients with HCC at BCLC substage B2 and preserved hepatic function. The primary endpoint was overall survival. A one-year survival rate threshold of 60% and an expected survival rate of 78%, based on previous reports of TACE, was assumed for setting the sample size. With a one-sided α-type error of 5% and 70% detection power, 25 patients were required over a 2-year enrollment period and 10-month follow-up period.

**Results:**

Thirty-one patients were enrolled in this study from June 2018 to June 2020. The 1-year survival rate was 71.0% (90% confidence interval, 68.4–73.6%). Median overall and progression-free survival periods were 17.0 and 10.4 months, and the objective response rates according to Response Evaluation Criteria in Solid Tumor (RECIST) version 1.1 and modified RECIST criteria were 22.6% and 70.0%, respectively. Common adverse events (AEs) were fatigue (68%), hypertension (65%), anorexia (61%), palmar-plantar erythrodysesthesia (39%), and thrombocytopenia (32%) of any grade; aspartate aminotransferase increased (23%), alanine aminotransferase increased (16%), and grade ≥ 3 proteinuria (13%). Treatment interruption and dose reduction were required in 61% and 81% of patients, respectively. LEN was discontinued in 29 patients due to disease progression (*n* = 17), AEs (*n* = 9), conversion to curative treatments (*n* = 2), and sudden death (*n* = 1), whereas post-LEN treatments were administered in 18 patients, including systemic chemotherapy (*n* = 11), TACE (*n* = 6), transarterial infusion (*n* = 1) and clinical trial (*n* = 1).

**Conclusions:**

The results suggest that LEN provides treatment benefits as an initial therapeutic in patients with BCLC substage B2 HCC with a safety profile comparable to that previously reported.

## Background

Primary hepatic cancer, frequently observed worldwide, is the sixth most common of all primary cancers and the third most common cause of cancer deaths [1, 2]. A similar aspect is seen in Japan, as > 30,000 individuals died of the disease in 2009. This was the fifth highest rate among all cancer deaths, following deaths from colon, lung, stomach, and pancreatic cancers [3]. In 2018, approximately 38,000 people in Japan were diagnosed with hepatic cancer [4]. Hepatocellular carcinoma (HCC), the most common type (94%) of hepatic cancer, is usually a consequence of chronic hepatitis or cirrhosis following persistent infections caused by hepatitis B virus (HBV), hepatitis C virus (HCV), or excessive and long-term alcohol consumption [5].

Various therapeutic methods are available to treat HCC depending on disease stage, physical status, and hepatic functional reserve in individual patients, including surgical resection, local puncture (radiofrequency ablation and percutaneous ethanol/acetic acid injection), liver transplantation, transcatheter arterial embolization including chemoembolization (TACE), transcatheter arterial infusion (TAI), and systemic chemotherapy, and the 5-year survival rate of HCC patients has improved considerably in recent decades [6]. However, concern persists that some patients may not be sufficiently treated with clinical benefits. Although TACE has been recommended as the first-line modality for HCC at the intermediate stage (Barcelona Clinic Liver Cancer [BCLC] stage B) [7], a variety of tumor burdens and hepatic functional reserves are associated with disease heterogeneity of this stage, raising skepticism about the broad utility of TACE [8–10].

To clarify treatment options that consider disease heterogeneity, stage subclassification (substages B1–B4) has been proposed using Child–Pugh score and Milan criteria–based up-to-seven criteria [8, 11–13], and alternatives to TACE or transarterial radioembolization are listed as potential first-line options (see Fig. 3 in Ref. [8]). If hepatic function deteriorates during conventional first-line TACE treatment, second-line systemic chemotherapy will not be applicable. TACE is contrarily applicable to patients with deteriorated hepatic functional conditions such as Child–Pugh class B. Thus, a versatile treatment modality will be applicable if systemic chemotherapy is applied first, followed by TACE.

Lenvatinib mesylate (LEN; Lenvima® capsule) is an orally acting antiangiogenic agent that inhibits vascular endothelial growth factor receptors 1–3, fibroblast growth factor receptors 1–4, platelet-derived growth factor receptor-α, and RET and KIT tyrosine kinases. It was first approved for the treatment of radioactive iodine–unresponsive differentiated thyroid cancer and then for treating advanced renal cell carcinoma as a second-line therapy in combination with everolimus. For the treatment of unresectable HCC, LEN was demonstrated in a phase 3 trial (REFLECT) for its non-inferiority versus sorafenib (SOR) in terms of overall survival (OS). The trial also demonstrated the clinical significance of LEN over SOR with regard to progression-free survival (PFS), time to progression, and objective response rate (ORR) [14]. A similar efficacy and safety profile of LEN was observed in a Japanese subset recruited by REFLECT [15]. Accordingly, LEN was approved for the treatment of unresectable advanced HCC in Japan in 2018.

Kudo et al. recently conducted a proof-of-concept study to compare the effectiveness of LEN and TACE. They analyzed albumin-bilirubin scores, OS, ORR, PFS, and safety and showed the superiority of LEN over TACE as first-line treatment for patients with intermediate-stage HCC [16]. This prospective observational study examined the usage, efficacy, and safety profile of LEN in Japanese patients with BCLC substage B2 HCC. While patients with substage B3 HCC are also subjects of this study, their enrollment is underway.

## Methods

### Patient cohort

Patients with BCLC substage B2 disease were included in this study. Substage B2 was defined as Child–Pugh score, 5–6; up-to seven criteria, Out (> 7); Eastern Cooperative Oncology Group-Performance Status (ECOG PS), 0; and no portal vein thrombosis (see Fig. 3 in Ref. [8]).

According to our judgment, patients meeting all of the following inclusion criteria were eligible for study registration but were excluded if any of the exclusion criteria were applicable. The inclusion criteria were as follows: patients with histologically and clinically^1^ confirmed HCC; diagnosis of BCLC substage B2; judged by the attending physician that the use of LEN was appropriate because locoregional therapy was not applicable; no history of treatment since confirmation of the present disease substage up to study registration and no prior systemic chemotherapy; aged 20 years or older at registration; recommended functional reserve of the major organs as well evidenced by laboratory tests within 14 days prior to registration; and scheduled for LEN monotherapy. Patients were excluded if they had uncontrolled hypertension; HCC invading the bile duct; had received any treatments for HCC within 28 days prior to registration; hemorrhagic symptoms due to ulcer, esophageal varices, etc. or thrombotic embolism such as brain infarction, cardiac infarction, deep venous thrombosis, or at high risk thereof; history of hepatic transplantation; diagnosis of hepatic encephalopathy; and active double cancer including synchronous cancer and metachronous cancer confirmed after a ≤ 3-year disease-free period.

### Patient registration

For an HCC patient judged eligible for the study (see patient cohort), the attending investigator obtained written informed consent. Thereafter, the investigator completed a study registration form and faxed it to the study administration office (S. Kobayashi, MD, the Division of Hepatobiliary and Pancreatic Oncology, Kanagawa Cancer Center) prior to the initiation of LEN. The patients were registered for study participation upon confirmation of their eligibility.

### Treatment with LEN and subsequent treatment

LEN (4 mg capsule) was started within 28 days post-registration at a dose of 12 mg or 8 mg once daily depending on body weight (≥ 60 kg or < 60 kg, respectively). A LEN dose reduction or interruption was allowed according to the investigator’s judgment depending on adverse events (AEs) occurrence in reference to the Japanese drug package insert. AEs were recorded for up to 30 days after the final use of LEN. The drug’s efficacy was monitored in each patient until death or final follow-up. Anti-cancer medications after LEN termination were not specifically defined but were allowed based on the investigator’s consideration.

### Tests and examinations

Dynamic CT or dynamic MR of intrahepatic lesions, lung field X-ray photography, and chest CT (when appropriate) were performed within 4 weeks prior to LEN initiation. Blood tests, including AFP and DCP determination, were performed within 2 weeks prior to LEN initiation. During the treatment period, laboratory tests, ECOG PS, blood pressure, patient’s subjective symptoms, and objective findings were recorded periodically. Abdominal CT or MR was recommended every 6 weeks.

### Outcomes

OS, the primary endpoint, was defined as the period from study registration to patient death of any cause. Patients who survived the study were censored at the time of final survival confirmation. A patient lost to follow-up was censored at the date of survival confirmation immediately prior to loss to follow-up. Tumor response to LEN was evaluated according to Response Evaluation Criteria in Solid Tumors (RECIST) version 1.1 [17] and modified RECIST (mRECIST) [18] based on tumor imaging findings and categorized as complete response (CR), partial response (PR), stable disease (SD), progressive disease (PD), and non-evaluable (NE). The ORR and disease control rate (DCR) were calculated as CR + PR and CR + PR + SD, respectively. PFS was calculated as the period during which a patient survived without disease progression. As mentioned earlier, AEs were collected from each patient during the study period, including 30 days after the final LEN administration. AE severity was graded as Grade 1–5 according to the Common Terminology Criteria for Adverse Events (CTCAE) version 4.0.

### Statistical points and analysis

The median OS and 1-year survival rates were generally comparable among published studies as 15.6–26.9 months and 59.2–75.5% for substage B2 [19–24]. Given these values as historical controls, the study’s statistical points are summarized below. We set the 1-year survival rate after TACE as 60% as a threshold and expected the use of LEN to further improve the rate to 78% for patients with substage B2 disease. Using Schoenfeld and Richter’s method [25], the required sample size was 25 according to the 2-year enrollment period and 10-month follow-up period, with a 70% detection power at a one-tailed significance level of 5%. With this planned sample size, we evaluated the efficacy (OS, median survival time, 1-year survival rate, PFS, and tumor response) and safety profile of LEN. Cox regression analysis was performed using the forced entry method to determine the potential prognostic factors by age, AFP level, baseline tumor size/number, and Child–Pugh score of the patients who received LEN.

## Results

### Patients’ baseline characteristics

As mentioned above, data obtained from patients with substage B2 disease are described in this report. Thirty-one eligible patients were enrolled from June 8, 2018, to June 7, 2020. The patients’ background variables at the time of the data cut-off (April 7, 2021) are summarized in Table 1.

**Table 1:** Baseline characteristics of the enrolled patients with hepatocellular carcinoma at substage B2

*N* = 31	n^a^ (% or range)
Age (years)	
Median (range)	77 (57–86)
Sex	
Male	29 (94)
Female	2 (6)
Body weight	
≥ 60 kg	17 (55)
< 60 kg	14 (45)
Etiology	
HBV	3 (10)
HCV	6 (19)
Alcohol	9 (29)
Other	13 (42)
Child–Pugh score	
5	20 (64)
6	11 (36)
Maximum diameter of liver tumor (mm)	
Median (range)	36 (10–135)
Liver tumor number	
< 10	23 (74)
≧10	8 (26)
Prior history of therapy	
Yes	14 (45)
No	17 (55)
Blood albumin (g/dL)	
Median (range)	3.9 (3.2–4.7)
Total bilirubin (mg/dL)	
Median (range)	1.0 (0.9–1.0)
Platelet count (× 10^4^ /μL)	
Median (range)	14.2 (6.4–37.8)
α-fetoprotein (ng/mL)	
Median (range)	52.1 (2.4–49,800)

^a^n is the number of patients corresponding to the category of each baseline item, or is the median value of each item. Numbers in parenthesis denote percentages or ranges depending on the variable attributes

The median patient age was 77 years (range, 57–86). The primary etiology of HCC was HBV, HCV, and alcohol consumption in 3, 6, and 9 patients, respectively, while it was unknown in 13 patients. The Child–Pugh score was 5 in approximately two-thirds of the 31 patients. More than 70% of the patients had fewer than 10 intrahepatic tumors. The maximum tumor diameter was 10–135 mm (median, 36 mm). Nearly half of the patients received prior treatment for HCC, including partial hepatectomy, radiofrequency ablation, percutaneous ethanol injection therapy, and TACE.

### Patient survival and tumor responses

**Fig. 1:**
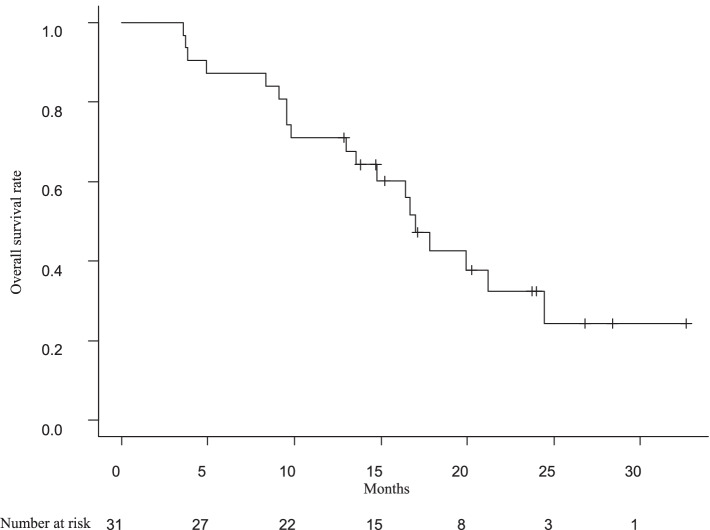
Kaplan–Meier curve of overall survival

The OS rate was plotted over time after registration (Fig. 1). The median OS was 17.0 months (95% CI, 15.3–19.2). The 1-year survival rate was 71.0% (90% CI, 68.4–73.6%) and the primary endpoint was met. The 2-year survival rate was 32.3% (95% CI, 13.2–51.4%).

**Fig. 2:**
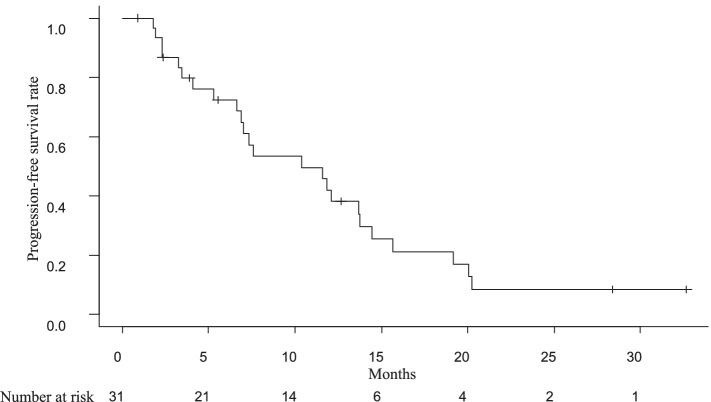
Kaplan–Meier curve of progression-free survival

The PFS rate was plotted over time after registration (Fig. 2). The median PFS was 10.4 months (95% CI, 6.6–13.8) and the 1-year PFS rate was 42.0% (95% CI, 23.5–59.4%).

The best tumor response to LEN is summarized in Table 2 according to RECIST 1.1 [17] and mRECIST [18]. One patient (3.2%) showed CR to LEN treatment using both evaluation methods. Only six (19.4%) were judged as PR by RECIST 1.1 versus 21 (67.7%) by mRECIST. Conversely, 21 (67.7%) and 6 (19.4%) were judged as SD by RECIST 1.1 and mRECIST, respectively. PD was observed in only three (9.7%) patients using either method. These values led to a 22.6% ORR and a 90.3% DCR according to RECIST 1.1 versus a 70.0% ORR and a 90.3% DCR according to mRECIST.

**Table 2:** Tumor response to lenvatinib according to RECIST 1.1 and mRECIST

Response	RECIST	mRECIST
	N (%)	N (%)
CR	1 (3.2)	1 (3.2)
PR	6 (19.4)	21 (67.7)
SD	21 (67.7)	6 (19.4)
PD	3 (9.7)	3 (9.7)
ORR	7 (22.6)	22 (70.0)
DCR	28 (90.3)	28 (90.3)

Best response of each patient was summarized in the table

*CR* complete response, *PR* partial response, *SD* stable disease, *PD* progressive disease, *ORR* objective response rate, *DCR* disease control rate

### Exposure and safety profile

Nineteen (61%) and 25 (81%) patients experienced LEN interruption and dose reduction, respectively. The median dose intensity of the drug was 5.0 mg/day (range, 0.9–12.0), while the relative dose intensity was 61.8% (range, 7.5–100.0%). The AEs are listed in Table 3. Fatigue (68%), hypertension (65%), anorexia (61%), proteinuria (45%), diarrhea (42%), aspartate aminotransferase (AST) increased (42%), and palmar-plantar erythrodysesthesia (PPES; 39%) were most frequently observed AEs. The frequently observed grade 3 or higher AEs were AST increased (23%), alanine aminotransferase (ALT) increased (16%), and proteinuria (13%). Thus, the overall safety profile of LEN was consistent with that observed in REFLECT with no new safety concerns [15, 17].

**Table 3:** Adverse events^a^

AE term	All grades	Grade ≥ 3
	**n (%)**	**n (%)**
Fatigue	21 (68)	3 (10)
Hypertension	20 (65)	3 (10)
Anorexia	19 (61)	2 (6)
Proteinuria	14 (45)	4 (13)
Diarrhea	13 (42)	0
AST increased	13 (42)	7 (23)
PPES	12 (39)	1 (3)
Thrombocytopenia	10 (32)	1 (3)
ALT increased	9 (29)	5 (16)
Bilirubin increased	8 (26)	3 (10)
Skin rash	6 (19)	0
Peripheral edema	6 (19)	0
Amylase increased	5 (16)	0
Hoarseness	5 (16)	0
Nausea	3 (10)	0
Abdominal pain	3 (10)	0
Dysgeusia	2 (6)	0
Mucositis oral	2 (6)	0

^a^AEs were collected up to 30 days following the final use of LEN. Percentage was calculated as the number of patients with AE divided by the whole patient population enrolled (*N* = 31)

*AST* aspartate aminotransferase, *PPES* palmar-plantar erythrodysesthesia, *ALT* alanine aminotransferase

### Post-treatment

At the time of the data cut-off, two (6%) patients were still receiving LEN treatment, while 29 (94%) had discontinued it. The reasons for discontinuation were disease progression (*n* = 17 [55%]), AE occurrence (*n* = 9 [29%]), tumor shrinkage (*n* = 2 [6%]), and sudden death (*n* = 1 [3%]). Of the 29 patients, 11 (38%) received no post-LEN anti-cancer therapies, 9 received only best supportive care, 1 reached CR, and 1 died suddenly of unknown causes after showing a PR to LEN. Eighteen (18 [62%]) received post-LEN anti-cancer therapies including TACE (6 [21%]), other systemic chemotherapies (11 [38%]) including SOR (*n* = 4)/ramucirumab (*n* = 4)/atezolizumab (ATZ; anti-programmed cell death 1 ligand 1 monoclonal antibody) plus bevacizumab (BEV; anti-VEGF monoclonal antibody) (*n* = 2)/other investigational drugs (*n* = 1), and TAI (1 [3%]).

### Subgroup analysis

To determine the prognostic factors in LEN-treated patients, a subgroup analysis was performed based on age (≥ or < 75 years), AFP level (≥ or < 400 ng/mL), tumor diameter (≥ or < 50 mm), liver tumor number (≥ or < 10), and Child–Pugh score (5 or 6). As seen in Table 4, Child–Pugh score was a key factor for prognosis among the patients treated with LEN, with median OS = 21.5 months (95% CI, 15.3–27.6) vs. 13.2 months (95% CI, 7.2–19.1) for those with a baseline Child–Pugh score of 5 and 6, respectively, which resulted in a hazard ratio of 3.206 (95% CI, 1.081–9.509).

**Table 4:** Multivariate sub-group analysis of prognostic factors

Factor	MST	Univariate analysis	Multivariate analysis		
		*p*-value	HR	95% CI	*p*-value
Age, years					
< 75 (ref) vs ≥ 75	16.9 vs. 17.3 m	0.653	2.191	0.690–6.954	0.183
AFP, ng/mL					
< 400 (ref) vs ≥ 400	18.1 vs. 9.9 m	0.490	1.921	0.528–6.986	0.322
Maximum diameter of liver tumors (mm)					
< 50 (ref) vs ≥ 50	20.2 vs. 13.7 m	0.129	1.479	0/537–4.077	0.449
Number of liver tumors					
< 10 (ref) vs ≥ 10	18.1 vs. 16.7 m	0.854	0.849	0.261–2.758	0.758
Child–Pugh score					
5 (ref) vs. 6	21.5 vs. 13.2 m	0.041	3.206	1.081–9.509	0.036

Forced entry model-based Cox regression method was employed for the multivariate sub-group analysis

*MST* median survival time, *HR* Hazard ratio, *CI* confidence interval, *AFP* α-fetoprotein

## Discussion

The prognosis of patients with intermediate-stage (BCLC stage B) HCC was generally comparable among subclasses (B1, B2, B3, and B4) in terms of median OS and 1-year survival rate [21–26]. Given these numbers as historical controls and the recent major progress in chemotherapy in patients with Child–Pugh class A functional reserve [14, 15], we conducted the present single-arm observational study. Thirty-one patients with BCLC substage B2 participated in the study, and their 1-year survival rate was 71.0% (90% CI, 68.4–73.6%), higher than 60%, an assumed threshold as the rate following TACE treatment, and close to 78%, which was expected for LEN in the sample size estimation. However, data from REFLECT and the updated IMbrave150 with ATZ plus BEV combination (a recently accepted first-line therapy; see below) showed a longer median OS of 18.5 months [14] and 19.2 months [26], respectively, in patients with BCLC B stage versus the 17.0 months obtained with LEN in the present study. The subgroup analysis presented here indicates that the Child–Pugh score is a critical factor for the prognosis of intermediate-stage HCC. Ando et al. and Hiraoka et al. also reported that the Child–Pugh score was critical for disease prognosis [27, 28]. The high proportion (36%) of patients with a Child–Pugh score of 6, versus ~ 22% in REFLECT [29] and ~ 28% in IMbrave150 [30], may have led to the shorter median OS in the present study.

The ORR was 70.0% by mRECIST and higher than that (40%) observed in REFLECT [14]. While the efficacy assessment was largely different depending on RECIST or mRECIST, for example, 22.6% vs. 70.0% for ORR in the present study, this was probably due to the mechanism of action of LEN, which decreased arterial flow to the tumors, and was likely assessed as positive by mRECIST. The DCR was also high (90.3%) irrespective of the evaluation criteria in the present study.

Given the ATZ plus BEV combination as a recently accepted first-line setting for the treatment of advanced HCC [26, 31], another study of the first-line use of the combination is ongoing in BCLC substage B2 HCC patients (study code: jRCTs071200051). The results of this study may further support the use of first-line systemic chemotherapy rather than TACE.

If systemic therapy is applied first at the stage with well-retained hepatic function, patients should be able to receive a step-by-step treatment modality, such as systemic therapy followed by TACE, depending on disease progression. However, of the 29 patients who discontinued LEN in the present study, only 6 (21%) received TACE, while 11 (38%) continued systemic therapy with other agents. The reason for the subsequent systemic therapeutic choice was likely due to retained hepatic functional reserve plus other reason(s), such as the appearance of distant metastasis, for which TACE was not applicable. The 1-year survival rate was 71%, while the 2-year survival rate was 32% with LEN, further suggesting that TACE may not be a key strategy for BCLC substage B2 HCC patients.

Despite the above considerations, TACE may still be useful when used concomitantly with systemic therapeutic agents. A clinical trial of a LEN-TACE combination is in progress, although the potential benefits of concomitant use of SOR and TACE have not been clearly evidenced [32–34] except for the results of TACTICS, which reported significantly longer PFS in the TACE plus SOR group than in the TACE alone group [35]. Multinational studies of the concomitant use of TACE with other systemic anti-cancer agents, such as ATZ plus BEV and immune checkpoint inhibitors, are currently ongoing. Sequential systemic therapeutic approaches were also reported using SOR and then the second chemotherapeutic agents such as regorafenib, ramucirumab, etc.[36–38], suggesting a potential expansion of chemotherapeutic choices for the HCC patients.

The present study used a non-randomized uncontrolled design with a small sample size; therefore, it was associated with data interpretation biases and had insufficient power to confirm the superiority of LEN over TACE, as represented by the observed 1-year survival rate (71%) over the threshold (60%) but below the expected value (78%). However, the overall results presented here, including the safety profile, were comparable to those of previous reports [15, 17], suggesting that LEN rather than TACE should preferably be used in BCLC substage B2 HCC patients. Our results support the use of a phase 3 randomized controlled trial to further confirm valid therapeutic options in intermediate-stage HCC patients.

Moreover, LEN is an oral ambulant agent that will be useful for patients compared to TACE, which requires hospitalization. Finally, it should be noted that the management of AEs with tailored SOR dosing was reported to prolong the survival of HCC patients [39], and this may simply be valid for the tailored use of LEN.

## Conclusions

LEN was efficacious in treatment-naïve BCLC B2 substage HCC patients compared to TACE, which was used in historical control studies. The safety profile of LEN was similar to that previously reported. These results suggest that LEN is preferable to TACE for the initial treatment of BCLC substage B2 HCC patients.

## Data Availability

The data that support the findings of this study are not publicly available because they contain information that could compromise the privacy of the research participants; however, they are available from the corresponding author (S. Kobayashi) upon reasonable request.

## Abbreviations

AE: Adverse event
AFP: α-Fetoprotein
ALT: Alanine aminotransferase
AST: Aspartate aminotransferase
ATZ: Atezolizumab
BCLC: Barcelona Clinic Liver Cancer
BEV: Bevacizumab
CR: Complete response
CT: Computed tomography
CTCAE: Common Terminology Criteria for Adverse Events
DCP: Des-gamma-carboxy prothrombin
DCR: Disease control rate
ECOG PS: Eastern Cooperative Oncology Group Performance Status
HBV: Hepatitis B virus
HCC: Hepatocellular carcinoma
HCV: Hepatitis C virus
LEN: Lenvatinib
mALBI: Modified albumin-bilirubin grade
MR: Magnetic resonance
NE: Non-evaluable
ORR: Objective response rate
OS: Overall survival
PD: Progressive disease
PFS: Progression-free survival
PPES: Palmar-plantar erythrodysesthesia
PR: Partial response
RECIST: Response Evaluation Criteria in Solid Tumors
SD: Stable disease
SOR: Sorafenib
TACE: Transcatheter arterial embolization
TAI: Transcatheter arterial infusion
VEGF: Vascular endothelial growth factor

## Footnotes

1 Abdominal contrast computed tomography (CT) or abdominal contrast magnetic resonance imaging (MR) was required to show an image of an early-phase tumor stain and late-phase washout, and the blood levels of either α-fetoprotein (AFP) or des-gamma-carboxy prothrombin (DCP) had to exceed the upper limit of the site-defined standard range.
